# The clinical features and risk factors of coagulopathy associated with cefoperazone/sulbactam: a nomogram prediction model

**DOI:** 10.3389/fphar.2024.1505653

**Published:** 2025-01-03

**Authors:** Changjing Xu, Junlong Zhu, Kun Tu, Hui Tang, Xinxin Zhou, Qiuyu Li, Kun Chen, Xuping Yang, Yilan Huang

**Affiliations:** ^1^ Department of Pharmacy, The Affiliated Hospital of Southwest Medical University, Luzhou, China; ^2^ Department of Vascular Surgery, The Affiliated Hospital of Southwest Medical University, Luzhou, China; ^3^ School of pharmacy, Southwest Medical University, Luzhou, China

**Keywords:** cefoperazone/sulbactam, coagulation disorders, risk factor, nomogram, predictive model

## Abstract

**Background:**

Cefoperazone/sulbactam (CPZ/SAM) is an important treatment option for infections caused by multidrug-resistant gram-negative bacteria. However, it is associated with an increased risk of coagulation disorders (CD) and causes severe bleeding in some instances. Early identification of risk factors and prediction of CD related to CPZ/SAM are crucial for prevention and treatment. This study aimed to explore the risk factors and developed a nomogram model for predicting the risk of coagulopathy in patients undergoing CPZ/SAM treatment.

**Methods:**

A total of 1719 patients who underwent CPZ/SAM in the Affiliated Hospital of Southwest Medical University from August 2018 to August 2022, were recruited as the training cohort. For validation, 1,059 patients treated with CPZ/SAM from September 2022 to August 2024 were enrolled. Patients were divided into the CD and the N-CD groups. The occurrence of CD was designated as the dependent variable. The univariate and multivariate logistic regression analysis was performed to identify the risk factors of CD. A nomogram model was constructed from the multivariate logistic regression analysis and internally validated for model discrimination and calibration. The performance of the nomogram was estimated using the concordance index (C-index) and calibration curve.

**Results:**

The multivariate logistic regression analysis resulted in the following independent risk factors for CD: baseline INR level (OR: 5.768, 95% CI: 0.484∼11.372, *p* = 0.036), nutritional risk (OR:2.711, 95%CI: 1.495∼4.125, *p <* 0.001), comorbidity of digestive system (OR:1.287, 95%CI: 0.434∼2.215, *p* = 0.004), poor food intake (OR:1.261, 95%CI: 0.145∼2.473, *p* = 0.032), ALB level (OR: −0.132, 95%CI: −0.229∼-0.044, *p* = 0.005) and GFR< 30 mL/min (OR: 1.925, 95%CI: 0.704∼3.337, *p* = 0.004). The internal validation confirmed the model’s good performance (C-index, 0.905 [95% CI: 0.864∼0.945]). The calibration plots in the nomogram model were of high quality. Validation further confirmed the reliability of the nomogram, with a C-index of 0.886 (95% CI: 0.832–0.940).

**Conclusion:**

The nomogram model facilitated accurate prediction of CD in patients undergoing CPZ/SAM. And this could potentially contribute to reducing the incidence of CPZ/SAM-associated CD and consequently improving patients’ outcomes.

## 1 Background

Cefoperazone/sulbactam (CPZ/SAM) is a therapeutic combination of the third-generation cephalosporin cefoperazone and the β-lactamase inhibitor sulbactam. It is widely used for treating moderate to severe infections at multiple body sites, such as respiratory tract infections, urinary tract infections, and intra-abdominal infections, due to its high efficacy and tolerability (Chen et al., 2021; Xin et al., 2013; Lan et al., 2021). In the context of the ongoing challenge faced by clinicians and medical institutions regarding the emergence and treatment of multidrug-resistant gram-negative bacteria infections, CPZ/SAM’s broad-spectrum activity is significant. It is effective against multidrug-resistant gram-negative bacteria, including extended-spectrum β-lactamase (ESBL)-producing *Escherichia coli*, ESBL-producing *Klebsiella pneumoniae*, and carbapenem-resistant *Acinetobacter baumannii* (Chang et al., 2018; Lai et al., 2019).

However, despite its general tolerability, increasing studies have indicated an association between CPZ/SAM and coagulation disorders (CD), which in some cases, can lead to severe bleeding (Wang et al., 2020; Cai et al., 2016; Hu, 2019). Two major mechanisms of coagulopathy associated with CPZ/SAM are as follows: (1) The chemical structure of CPZ contains an N-methylthio-tetrazole (NMTT) side chain, which can inhibits the vitamin K-dependent step in clotting factor synthesis (Lipsky, 1983; Lipsky, 1988). (2) Cefoperazone is not metabolized significantly and approximately 85% of the dose is excreted into the biliary tract (Greenfield et al., 1983), which can largely kill vitamin K-producing intestinal bacteria (Lipsky, 1988; Mulligan et al., 1982).

Given these potential adverse effects, early identification of risk factors and prediction of coagulation dysfunction related to CPZ/SAM are crucial for prevention and early intervention. However, limited research has been conducted on the risk factors, and no reliable models are currently available to predict the risk of coagulopathy in patients receiving CPZ/SAM treatment. A nomogram, which visually represents the prognostic strength of various factors from a multivariate model in a single diagram, can offer highly accurate risk estimation. This tool allows clinicians to standardize clinical decision-making based on evidence and personalize it for each individual (Shariat et al., 2009; Balachandran et al., 2015). Therefore, the objective of this study was to retrospectively analyze risk factors and develop a nomogram model to predict the risk of CD in patients undergoing CPZ/SAM treatment.

## 2 Methods

### 2.1 Study subjects

This retrospective case-control study analyzed the clinical data of patients aged 18 years and above who received CPZ/SAM (cefoperazone: sulbactam, 2:1) treatment from the Affiliated Hospital of Southwest Medical University. This hospital is a major academic tertiary hospital with 4,200 beds and more than 130,000 inpatients annually (Luo et al., 2022). Patients who met the above conditions were assigned to the training group from August 2018 to August 2002. A separate test cohort was recruited from patients who attended the same medical center from September 2022 to August 2024 to validate the reliability of the prediction model. The detailed patient data were retrieved from the hospital information system.

### 2.2 Definitions

Each patient receiving CPZ/SAM was evaluated for the causal association between the drug and coagulopathy adverse drug reactions (ADRs) using the Naranjo algorithm, which consists of ten questions and is scored as follows: scores between 9 and 10 indicate definite ADRs; 5–8, probable ADRs; 1–4, possible ADRs; and less than 1, doubtful ADRs (Naranjo et al., 1981; Son et al., 2011). In this study, cases meeting the first three selection criteria were adjudicated as coagulopathy associated with CPZ/SAM. Common Terminology Criteria for Adverse Events (CTCAE) Version 5.0 was performed to evaluate the severity of coagulopathy ADRs. Serious ADRs were defined as CTCAE grade 3 or greater toxicities.

All of the patients had normal coagulation tests and no bleeding before CPZ/SAM therapy. We excluded patients who were concurrently receiving drugs affecting coagulation, including anticoagulants, anti-platelet agents, fibrinolysis or procoagulant drugs. Based on the occurrence of whether coagulation disorders (CD) after CPZ/SAM treatment, patients were divided into CD and the N-CD groups. For the CD group, the following criteria needed to be met: 1) The patients were adults over 18 years of age; 2) During the treatment, the indications, usage and dosage of CPZ/SAM were completely in accordance with the drug label and instruction; 3) CPZ/SAM treatment for more than 2 days; 4) Patients with complete medical records; 5) Patients without the basic diseases of blood and blood-forming organs, such as hemophilia; 6) Coagulation function was evaluated before, during, and after treatment. The study was approved by the Ethics Committee of The Affiliated Hospital of Southwest Medical University (No.KY2022030), all methods used in this study were performed in accordance with relevant guidelines and regulations. And informed consent was not required for the retrospective analysis of patient data.

### 2.3 Data collection

Predictors were pre-set by clinical expertise and literature review. All predictors were assessed on the day before the start of CPZ/SAM. A pre-designed form was created to collect clinical data, including general information (age, sex, recent food intake), daily dose and duration of CPZ/SAM use, laboratory (international normalized ratio (INR), activated partial thromboplastin time (APTT), fibrinogen, albumin (ALB), alanine transaminase (ALT), total bilirubin (TSB)) and glomerular filtration rate (GFR, Severe renal dysfunction was defined as a GFR less than or equal to 30 mL/min/1.73 m^2^). Nutritional risk was assessed using the Nutritional Risk Screening 2002 (NRS-2002), with a total score above 3 indicating nutritional risk (Kondrup et al., 2003). Comobidities of digestive system included malignant tumors, gastroenteritis, inflammatory bowel disease, Crohn’s disease, ulcerative colitis, peptic ulcer, acute pancreatitis, acute cholecystitis, acute cholangitis and so on.

### 2.4 Statistical analysis

Continuous variables were described using medians with interquartile range (IQR) or means with standard deviations (SD), while categorical variables were summarized as frequencies and percentages (n, %). For statistical analysis, χ2 test or Fisher exact test was used. Based on the univariate analysis results, multivariate logistic regression was conducted to identify independent risk factors for coagulation disorders. The effect of individual predictors on CD was reported as odds ratios (OR) with corresponding 95% confidence intervals (95%CI). We then used these significant independent factors to construct a nomogram for predicting coagulation disorders. The model’s discriminative ability was assessed using the concordance index (C-index), which is analogous to the commonly reported area under the receiver operating characteristic curve (AUC). A C-index of 1 indicated perfect concordance; The C-index of most models is 0.7–0.85 (Balachandran et al., 2015), while the model’s calibration was evaluated with a calibration curve. A DeLong test was used to evaluate the AUC difference between training set and test set. A two tailed *p*-value < 0.05 was considered statistically significant. Statistical analyses were performed in R version 4.3.6 (http://www.r-project.org/).

## 3 Results

### 3.1 Characteristics of the study population

**FIGURE 1:**
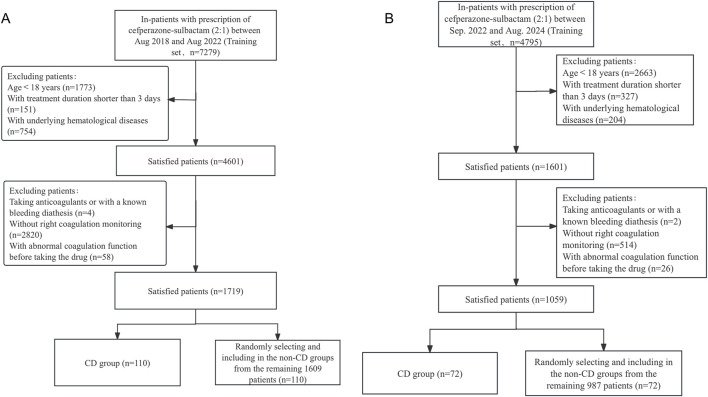
The flowchart of patient selection in the **(A)** training set and the **(B)** test set. CD, coagulation disorders.

7,279 infected patients were treated with CPZ/SAM (cefoperazone: sulbactam, 2:1) between August 2018 and August 2022, a total of 1719 patients who met the inclusion and exclusion criteria were divided into the training set. Among the selected patients, 110 (6.40%) exhibited CPZ/SAM associated CD and were assigned to the CD group. From the remaining 1,609 patients, 110 were randomly selected and included in the N-CD group using an Excel-generated random number table. The test set included 1,059 patients recruited based on the same criteria, with 72 (6.8%) developing CPZ/SAM-associated CD. The patient selection process was presented in Figures 1A, B.

The demographic and clinical data of the training and test cohorts were summarized in Table 1. The training set consisted 220 patients with an average baseline INR of 1.08 ± 0.093, 35.91% were aged ≥70years; 56.82% were male; 74.55% had nutritional risk. The test set comprised 144 patients with an average baseline INR of 1.06 ± 0.092, 39.58% were aged ≥70years; 61.11% were male; 65.97% had nutritional risk. Apart from the albumin level, there were no statistically significant differences in the characteristics between the two sets.

**TABLE 1:** Characteristics of the study population and comparison of the training set and test set.

Variables	Training set (n = 220)	Test set (n = 144)	*p*-value
Age≥70 years, %	79 (35.91)	57 (39.58)	0.55
Male, %	125 (56.82)	88 (61.11)	0.481
Baseline INR	1.08 ± 0.093	1.06 ± 0.092	0.078
Baseline APTT (s)	31.12 ± 4.45	30.2 ± 4.52	0.436
Nutritional risk, %	164 (74.55)	95 (65.97)	0.10
Comorbidity of the digestive system, %	84 (38.18)	64 (44.44)	0.28
Poor food intake, %	169 (76.82)	114 (79.17)	0.691
Daily dose>6g, %	148 (67.27)	96 (66.67)	0.995
Treatment for ≥7 days, %	143 (65.00)	89 (61.81)	0.611
ALT>40U/L, %	56 (25.45)	36 (25.00)	1.00
TSB>34.2umol/L, %	20 (9.09)	16 (11.11)	0.651
GFR<30 mL/min, %	35 (15.91)	21 (14.58)	0.846
ALB (g/L)	31.13 ± 4.39	31.94 ± 5.17	0.024^*^

Abbreviations: INR, international normalized ratio; APTT, activated partial thromboplastin time; ALT, alanine transaminase; TSB: total bilirubin; GFR: glomerular filtration rate; ALB, albumin. ^*^ *p*-value < 0.05.

### 3.2 Summary of ADRs of training set

Coagulopathy ADRs occurred between 3 and 16 days after the initiation of CPZ/SAM treatment in training set, with a median onset of 7 days (interquartile range [IQR], 5–10). Among 110 patients with coagulopathy ADRs, 96 scored between 5 and 8 points on Naranjo algorithm were are rated as probable ADRs, 14 scored between 1 and 4 points were possible ADRs. Most ADRs were mild or moderate; however, 47 were classified as serious according to CTCAE Version 5.0. Overall, 104 cases (94.55%) exhibited prolonged INR, 41 (37.27%) showed prolonged APTT, 8 (7.27%) had decreased fibrinogen levels, and 20 (18.18%) experienced bleeding.

ADRs in 103 patients were successfully treated by discontinuing CPZ/SAM, administering intramuscular vitamin K1, and infusing fresh frozen plasma. For the remaining 7 patients, their families opted to discontinue further treatment due to serious underlying diseases, and one of them died in the hospital.

### 3.3 Univariate analysis of the occurrence of coagulopathy ADRs in patients receiving CPZ/SAM

All variables listed in Table 2 were used for univariate analysis. The univariate analysis results showed that there was no significant difference in gender between the CD group and N-CD group (*p* > 0.05). The CD group showed a higher likelihood of being aged 70 years or older, being at nutritional risk, having comorbidities of the digestive system, having poor food intake, receiving a daily dose of CPZ/SAM greater than 6g and receiving CPZ/SAM for more than 7 days (*p* < 0.05). The comparison of laboratory data found that there was no statistically significant difference in baseline APTT, ALT and TSB levels between the two groups (Table 2). However, ALB and GFR levels were significantly lower than those of N-CD group (*p <* 0.05). What’s more, the baseline INR in CD group was significantly higher than that in N-CD group.

**TABLE 2:** The results of Univariate analysis of training set and test set.

Variables	Training set			Test set		
	CD (n = 110)	N- CD (n = 110)	*p*-value	CD (n = 72)	N- CD (n = 72)	*p*-value
Age≥70 years, %	47 (42.73)	32 (29.09)	0.049^*^	35 (48.61)	22 (30.56)	0.041^*^
Male, %	66 (60)	59 (53.64)	0.058	41 (56.94)	47 (65.28)	0.393
Baseline INR	1.11 ± 0.11	1.05 ± 0.07	<0.001^*^	1.09 ± 0.10	1.03 ± 0.08	<0.001^*^
Baseline APTT (s)	31.84 ± 4.45	30.06 ± 4.78	0.09	30.56 ± 4.62	29.84 ± 4.39	0.35
Nutritional risk, %	106 (96.36)	58 (52.73)	<0.001^*^	61 (84.72)	34 (47.22)	<0.001^*^
Comorbidity of the digestive system, %	53 (48.18)	31 (28.18)	0.002^*^	40 (55.56)	24 (33.33)	0.012^*^
Poor food intake, %	103 (93.64)	66 (60)	<0.001^*^	66 (91.67)	48 (66.67)	<0.001^*^
Daily dose>6g, %	82 (74.55)	66 (60)	0.031^*^	55 (76.39)	41 (56.94)	0.022^*^
Treatment for ≥7 days, %	82 (74.55)	61 (55.45)	0.0045^*^	51 (70.83)	38 (52.78)	0.040^*^
ALT>40U/L, %	30 (27.27)	26 (23.64)	0.642	19 (26.39)	17 (23.61)	0.847
TSB>34.2umol/L, %	13 (11.82)	7 (6.36)	0.241	7 (9.72)	9 (12.5)	0.791
GFR<30 mL/min, %	28 (25.45)	7 (6.36)	<0.001^*^	17 (23.61)	4 (5.56)	0.005^*^
ALB (g/L)	31.13 ± 4.41	35.19 ± 4.46	<0.001^*^	29.75 ± 4.97	34.14 ± 4.37	<0.001^*^

Abbreviations: CD, coagulation disorders; INR, international normalized ratio; APTT, activated partial thromboplastin time; ALT, alanine transaminase; TSB: total bilirubin; GFR: glomerular filtration rate; ALB, albumin. ^*^ *p*-value < 0.05.

### 3.4 Multivariate regression analysis of the occurrence of CD in patients receiving CPZ/SAM

All significant variables from the univariate analysis were considered as independent variables, and whether CD occurred was considered as dependent variable, then they were included in the multivariate logistic regression analysis (Table 3). The results showed that baseline INR level (OR: 5.768, 95% CI: 0.484∼11.372, *p* = 0.036), nutritional risk (OR:2.711, 95%CI: 1.495∼4.125, *p <* 0.001), comorbidity of digestive system (OR:1.287, 95%CI: 0.434 ∼2.215, *p* = 0.004), poor food intake (OR:1.261, 95%CI: 0.145∼2.473, *p* = 0.032), ALB level (OR: −0.132*,* 95%CI: −0.229∼-0.044, *p* = 0.005) and GFR < 30 mL/min (OR: 1.925*,* 95%CI: 0.704∼3.337, *p* = 0.004) were the independent risk factors influencing the occurrence of CD in patients receiving CPZ/SAM.

**TABLE 3:** The results of Multivariate logistic regression analysis of training set and test set.

Variables	Training set		Test set	
	OR (95%CI)	*p*-value	OR (95%CI)	*p*-value
Age≥70 years	0.098 (−0.723∼0.913)	0.813	0.908 (0.069∼1.943)	0.074
Baseline INR	5.768 (0.484∼11.372)	0.036^*^	6.36 (0.998∼12.045)	0.023^*^
Nutritional risk	2.711 (1.495∼4.125)	<0.001^*^	1.533 (0.479∼2.686)	0.006^*^
Comorbidity of the digestive system	1.287 (0.434∼2.215)	0.004^*^	1.572 (0.533∼2.735)	0.005^*^
Poor food intake	1.261 (0.145∼2.473)	0.032^*^	1.791 (0.547∼3.216)	0.008^*^
Daily dose>6g	0.693 (−0.164∼1.581)	0.117	0.815 (−0.218∼1.897)	0.128
Treatment for ≥7 days	0.740 (−0.111∼1.622)	0.092	0.811 (−0.215∼1.889)	0.127
ALB (g/L)	−0.132 (−0.229∼-0.044)	0.005^*^	−0.201 (−0.332∼-0.091)	0.001^*^
GFR<30 mL/min	1.925 (0.704∼3.337)	0.004^*^	1.639 (0.275∼3.259)	0.028^*^

Abbreviations: INR, international normalized ratio; CPZ/SAM, cefoperazone/sulbactam; GFR: glomerular filtration rate; ALB, albumin. ^*^ *p*-value < 0.05.

### 3.5 The nomogram of CD occurrence and the performance evaluation of the nomogram

**FIGURE 2:**
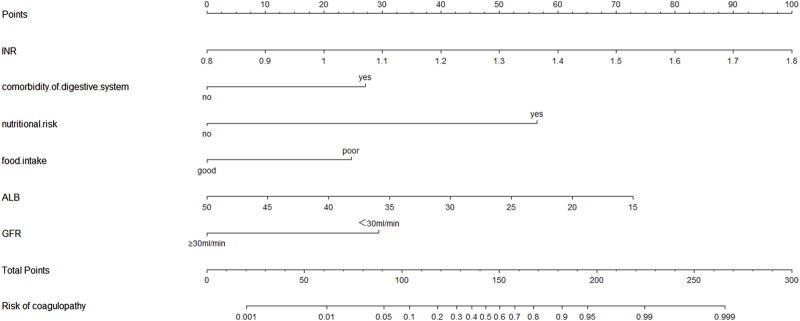
The nomogram for calculating the risk score and predicting the risk of CD in patients receiving CPZ/SAM treatment. To calculate total score and predicted probability of CD, points from individual variables are added and a vertical line is drawn from the total points line at the bottom downward to determine the predicted probability of CD due to CPZ/SAM. Abbreviations: CD, coagulation disorders; CPZ/SAM, cefoperazone/sulbactam; INR, international normalized ratio; CPZ/SAM, cefoperazone/sulbactam; GFR: glomerular filtration rate, mL/min; ALB, albumin, g/L.

**FIGURE 3:**
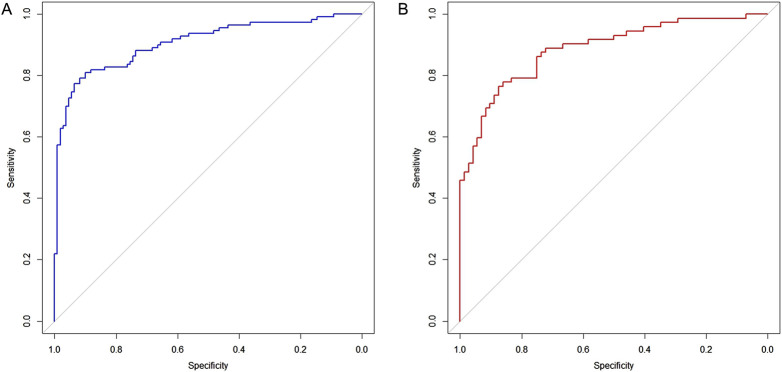
Receiver operating characteristic curves for evaluating the discrimination capability of plasma NSE and the final model. **(A)** Training set, AUC = 0.905 (95% CI: 0.864∼0.945). **(B)** Test set, AUC = 0.886 (95% CI: 0.832∼0.94).

**FIGURE 4:**
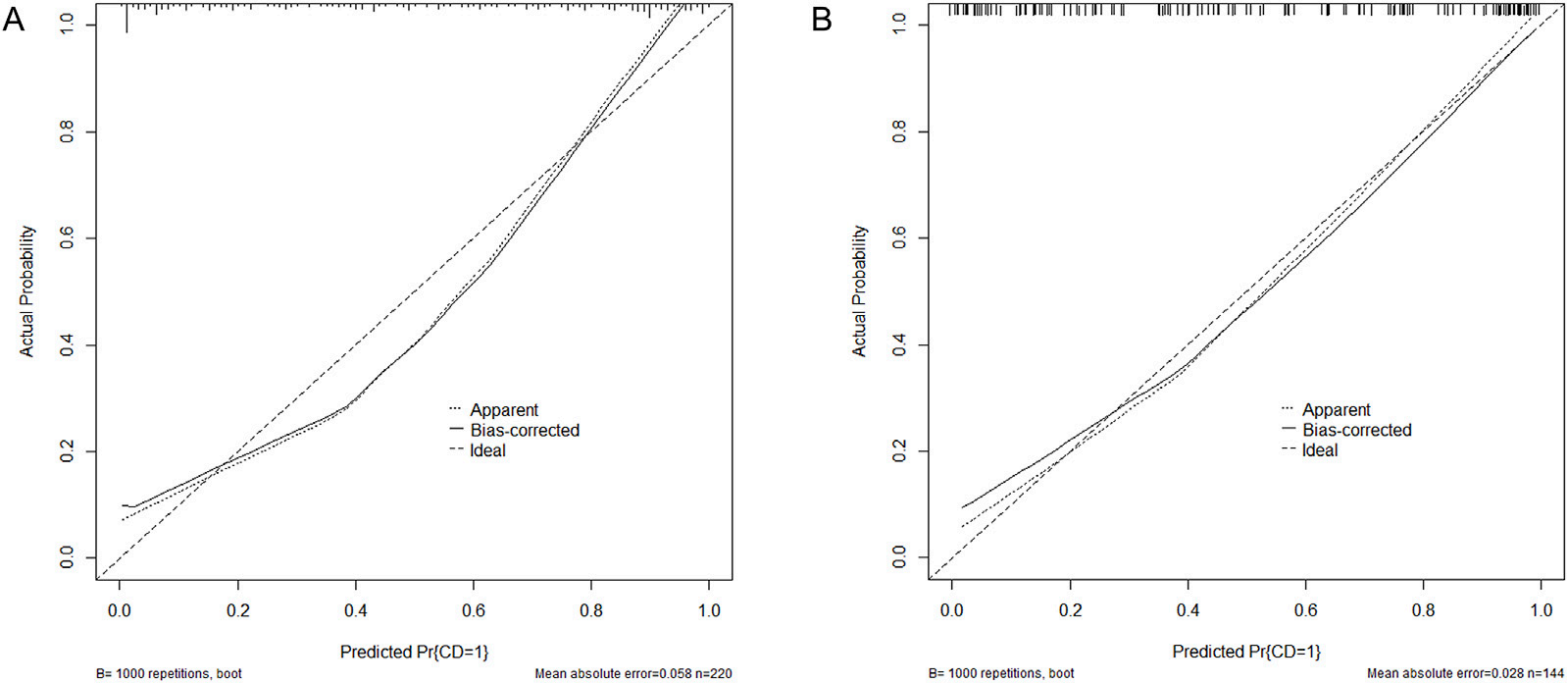
Calibration curves for the nomogram indicating good agreement between the predictors and observations. **(A)** Training set. **(B)** Test set. Pr, probability; CD, Coagulation disorders.

The six factors obtained from multivariate logistical regression analysis were used to establish a nomogram of the risk of CD (Figure 2). The ROC curve of prediction model was presented in Figure 3A, with an AUC of 0.905 (95% CI: 0.864∼0.945), indicating high prediction accuracy. In the test set, the AUC was 0.886 (95% CI: 0.832∼0.94) (Figure 3B). According to the DeLong test, the ROC AUC difference between training set and test set was not significant (p = 0.574). To verify the accuracy of the nomogram, internal validation was conducted using the bootstrap method with 1,000 resamples. Furthermore, the calibration curves in both sets demonstrated a high level of agreement between the predicted and ideal models (Figures 4A, B).

## 4 Discussion

CPZ/SAM is widely used for the treatment of gram-negative bacilli infections (Ku and Yu, 2021). However, due to its unique chemical structure and pharmacokinetic characteristics, its treatment may be associated with a high risk of CD (Wang et al., 2020). In response to this, China’s National Medical Products Administration requested modifications to drug labels in 2019 to include warnings about CD and bleeding risks. Despite the recognized risks, there have been few studies reported on the risk factors of CD in patients receiving CPZ/SAM. Thus, in this study, we used univariate analysis and multivariate logistic regression analysis to identify the risk factors for CD. Furthermore, we constructed a predictive nomogram to estimate the risk of CD for each patient treated with CPZ/SAM comprehensively and accurately.

Coagulopathy ADRs occurred at a median of 7 days (ranging from 3 to 16 days) after the start of CPZ/SAM treatment in this study, which was in agreement with prior report. Shao et al. (2023) showed that coagulopathy occurred at days 2–19 (7.380 ± 4.628) after administration of CPZ/SAM. Large amounts of CPZ in the gastrointestinal tract apparently alters vitamin K-producing gut flora, which may be the main cause of CD. This was confirmed by previous studies (Triger et al., 1988; Goss et al., 1992), they showed that treatment with other antibiotics containing a NMTT chain (e.g., cefotetan) did not significantly increase the risk of CD. In the present study, prothrombin time results are expressed as INR (Lee et al., 2017), CD in patients receiving CPZ/SAM was mainly presented by an elevated INR, but rarely bleeding. This phenomenon was consistent with previous research findings. Wang et al. (2020) and Strom et al. (1999) found that cefoperazone was associated with a higher risk of PT prolongation.

In the study, we determined 6 factors related to the risk of CD in patients receiving CPZ/SAM through univariate and multivariate regression analysis, including baseline INR level, nutritional risk, comorbidity of digestive system, poor food intake, ALB level and GFR < 30 mL/min, all of which were used to establish a nomogram of the risk of PO. As far as we know, this was the first nomogram study for assessing the risk of CD in patients receiving CPZ/SAM. And based on AUC and calibration curve evaluation, the nomogram model showed good accuracy and consistency.

This study showed that baseline INR level, comorbidity of digestive system, poor food intake, nutritional risk, ALB level and GFR < 30 mL/min were independent risk factors for CD in patients receiving CPZ/SAM. It was observed that patients receiving CPZ/SAM with higher INR baseline level were more likely to develop CD. In some cases, the INR is considered as a vitamin K-dependent parameter, for example, patients on chronic warfarin therapy require close laboratory monitoring of their INR (Barnes et al., 2018). Thus, CD is more likely to occur when INR baseline level is high in patients receiving CPZ/SAM.

It’s known that Vitamin K in the body is mainly obtained from diet and intestinal microbiome (Suttie and Booth, 2011; Zhang et al., 2021). Patients with comorbidity of digestive system often have inadequate absorption of vitamin K, and previous researches have also confirmed that malabsorption-related diseases resulted in vitamin K deficiency, like inflammatory bowel disease and primary biliary cholangitis (Nowak et al., 2014; Kuwabara et al., 2009; EASL Clinical Practice GuidelinesEuropean Association for the Study of the Liver, 2017). Therefore, poor food intake and comorbidity of digestive system contributed to vitamin K deficiency in patients receiving CPZ/SAM. Moreover, patients at nutritional risk were more likely to develop CD when receiving CPZ/SAM, and the mechanism was likely related to the inhibition of intestinal flora that synthesized vitamin K. The nutritional status of host affect the composition of the intestinal flora, simultaneously, changes in the gut nutritional environment and metabolic profile can markedly affect the activity of the gut microbiota (Dai et al., 2020; Fletcher et al., 2021).

In patients with hypoalbuminemia, the unbound proportion of highly protein-bound drugs increases due to the decrease in available binding sites. Furthermore, hypoalbuminemia is likely to increase the clearance (CL) of a drug (Ulldemolins et al., 2011; Parker et al., 2015). CPZ is a highly protein-bound drug with a binding rate of up to 90% (Peterson et al., 1989), it is excreted more quickly into the intestines through the biliary tract in patients with hypoalbuminemia. This action can affect the intestinal flora and maintenance of vitamin K. Gudivada KK et al. also showed that hypoalbuminemia was the risk factor for CPZ/SAM induced coagulopathy (Gudivada et al., 2023). CPZ is mainly excreted through feces (>75%), with less than 25% typically excreted through the kidneys. Previous study have indicated that no significant difference was observed in mean serum levels, half-life, and serum clearance between the groups with normal and severely impaired renal function (Greenfield et al., 1983). This may be because compared to normal patients, CPZ was excreted more via the feces in patients with severely impaired renal function when receiving an equivalent dose of CPZ/SAM, thus affecting the intestinal microecology.

Previous studies have suggested that the administration of vitamin K1 can somewhat reduce the occurrence of CD associated with CPZ/SAM (Shao et al., 2023; Wu et al., 2021; Ebid et al., 2024). However, research conducted by Gudivada KK et al. found that prophylactic vitamin K did not offer any benefit toward preventing INR elevation (Gudivada et al., 2023). In the current study, we also aimed to examine the preventive effects of prophylactic vitamin K on CD in patients receiving CPZ/SAM. Regrettably, few patients took vitamin K prophylaxis while taking CPZ/SAM in our unit. This will be further evaluated in our prospective study. However, given the inconsistent conclusions of previous studies, and the current model has demonstrated good prediction effects, we therefore do not think that this will have a major impact on the final model.

Our study has several strengths. Firstly, we are the first to perform a multivariate logistic regression analysis to explore independent risk factors for CPZ/SAM associated with CD. Most previous studies were case reports and univariate analysis. Secondly, we developed a simple and visually effective prediction nomogram model for the first time, which contains six factors that affect the occurrence of CD in patients receiving CPZ/SAM. The concordance index (C-index) was used to assess the performance, which was graded as very good (0.80–1.00) (Luo et al., 2019). In the current study, the nomogram demonstrated good performance for prediction (C-index of 0.905), which may improve and provide new insights for the early identification and intervention of CD in patients receiving CPZ/SAM. On the other hand, for patients with more than two risk factors or an anticipated CD risk greater than 10%, we recommend more frequent monitoring of coagulation indicators or using alternative antibacterial drugs with less impact on coagulation to minimize the risk of bleeding.

This study still has some limitations. Firstly, this retrospective study was conducted based on reviewing medical records from a single institution and the sample size was relatively small. Secondly, the model was internally validated with good accuracy and reliability, and external validation was still necessary. Finally, due to missing data, some potentially meaningful factors, such as prophylactic vitamin K, were not included in the risk factor analysis. It is necessary to validate our results with those of other centers. In the future, a prospective multi-center in-sample study to further confirm the reliability of the nomogram is warranted.

## 5 Conclusion

In conclusion, we constructed and validated a novel prognostic nomogram to predict the risk of CPZ/SAM induced CD. Through this model, clinicians could potentially identify patients at a higher risk of developing CD early on, allowing for timely intervention and management. This could lead to a reduction in the occurrence of CD associated with CPZ/SAM, and consequently improve patients’ outcomes.

## Data Availability

The original contributions presented in the study are included in the article/supplementary material, further inquiries can be directed to the corresponding authors.

## Abbreviations

CPZ/SAM, cefoperazone/sulbactam; CD, coagulation disorders; NMTT, N-methylthio-tetrazole; ADRs, adverse drug reactions; CTCAE, Common Terminology Criteria for Adverse Events; INR, international normalized ratio; APTT, activated partial thromboplastin time; ALT, alanine transaminase; TSB, total bilirubin; ALB, albumin; GFR, glomerular filtration rate; IQR, interquartile range; SD, standard deviation; OR, odds ratios; 95%CI, corresponding 95% confidence intervals; C-index, concordance index; AUC, receiver operating characteristic curve.
